# Selections and Abstracts

**Published:** 1899-09

**Authors:** 


### SELECTIONS AND ABSTRACTS.

### The Pathology and Therapy of Cancer, with Special Reference to Cancer of the Stomach.*

*Mr. President and Gentlemen:*

You will allow me to thank you for the high honor which you have conferred on me on this occasion. While your selection has done me great honor, the choice of subjects which would be of interest to the members of this representative body of professional men, is one which has caused me some anxiety. After ripe deliberation I have come to the conclusion that this is an opportunity for presenting the modern standpoint of a certain department of medical science. I have been engaged in practical surgical work for nearly twenty-five years as teacher and operator, and shall base my critical remarks on the advances that have been made in the treatment of cancer largely upon my own observations. There

* An Address delivered before the Oregon State Medical Society at its Annual Meeting at Portland, June, 1899. By Augustus C. Bernays, M.D., St. Louis, Mo., U. S. A.

has been great activity in the study of malignant neoplasms, and the last few years have brought about a marked change in the therapy of these diseases. We are now doing operations of such magnitude and extent and are invading regions not thought of formerly. In fact these operations in cancer cases are of daily occurrence, and are done by many surgeons in all parts of the world as a matter of principle and of duty. In an address of this kind it cannot be my object to present the results of original research so much as to present to you the present views, as they have been evolved by many laborers in the laboratory and in the clinic. The main object must be to arrive at some definite conclusions as to the value of our methods of managing the very common and apparently increasing numbers of cases of cancer that seek our advice and our aid.

I shall use the word cancer in its general sense, including the two forms of malignant neoplasms known as carcinoma and sarcoma. I have a right to assume that the histological structure of these growths is well known to all of you as presented in the modern text-books, and shall only call your attention to the fact that there are forms of sarcoma which so closely resemble some forms of carcinoma, that a differential diagnosis is not so simple as it might appear from the teachings of the text-books. Some very important investigations recently made seem to show that even epithelial growths are preceded by changes in the connective tissue which always underlies the epithelial layers of the skin and mucous membranes. I have myself seen two large-celled sarcomata which had a plain alveolar structure and puzzled all investigators very much indeed. I wish to go on record as holding that the sharp line which has been drawn between sarcoma and carcinoma cannot be maintained, and that in my opinion we are far from an understanding of the biological significance of the malignant tumors. The line of research which promises finally to solve the question as to the origin of tumors has been followed in a most laudable but as yet only tentative way by that brilliant English surgeon, Mr. Bland Sutton. His little work on tumors is so instructive and so concisely written that I cannot refrain from recommending it to you, and promise you a rare treat from its perusal. While speaking on the literature I can say that the work on tumors by my friend, Nicholas Senn, of Chicago, is the only work on the subject written by an American surgeon which deserves mention. It is a truly great book, and stands next in importance to Senn's principles of surgery among the works of that admirable and indefatigable surgeon.

I have just stated that a sharp line cannot be drawn between sarcoma and carcinoma, and I must now tell you that a line cannot be drawn which will everywhere separate malignant from benign tumors. It is a fact that the microscope alone cannot be relied upon to make a differential diagnosis between sarcoma and some forms of granulomata. Sarcoma cannot always be distinguished from lymphoma, nor from gumma, and not even from some tubercular tumors, by any known histological methods. The absence of tubercle bacilli in a tumor does not prove that tubercle bacilli are not the producers of toxin which may have caused the proliferation of cells in question. Even when to the history and the microscopical examination of sections of a tumor is added a full clinical examination by means of all our modern methods and by means of all our instruments of precision a reliable diagnosis is sometimes impossible. Fortunately for us and for our patients, these cases are extremely rare, but I felt constrained to bring out the existing defect in this address because we are strongest when we best appreciate our weaknesses.

Along the line of bacteriological research all attempts to solve the question as to the etiology of tumors have not advanced our knowledge much. The numerous alleged discoveries recently published still await corroboration, and while no doubt some valuable facts have been discovered, their authors have failed to prove that the various parasites, some resembling amœba, others belonging to the coccidia, and still others closely resembling ordinary fungi of decomposition and saprophytes, are really etiological or causative in the development of cancer. These workers have, however, succeeded in proving to us that under the head of what we call cancers, malignant neoplasms, some widely different things are included. When we think of a malignant papilloma or wart, of a small black sarcoma of the choroid, beautifully visible in the background of the eye, of a soft almost bloodless, white or gray sarcoma of the fascia lata; again, of a hard scirrhous tumor of the breast or of a pulsating blue vascular sarcoma of the orbit, we must all believe that under the head of cancer are gathered a great many different things which probably are etiologically widely different, and we all know that our notion of malignancy is a most elastic one. We know that some of these malignant growths are very slow in doing their deadly work while others kill almost like acute infections caused by virulent poisons or toxins.

While I am convinced from my own observations and experiments that some forms of cancer are purely of extraneous or parasitic origin, I am convinced that the great majority of tumors, benign as well as malignant, are developmental in their origin. It will be shown that the large majority are referable to some ancestral inherited defect. The vast majority of tumors will be found to be traceable to a rudiment of an organ which, ages ago, played a more or less important part in the physiology of our progenitors. Drawing the logical conclusion from the facts that have been proven about the descent of man from forms of life, which in a long unbroken line have been developed on this planet since it cooled off to such a degree as to permit the existence of life as we know it, we can truly say that we have almost infinite atavistic possibilities. Just as we now positively know that a congenital fistula of the neck is a remnant of a branchial cleft, of a gill, as it were, so will we some time in the future know how to explain the thousands of deformities and neoplasms, malignant as well as benign. The solution of the question as to the origin of tumors will come when the ontogeny and the phylogeny of the human animal will have been worked out to a high degree by morpho logical, embryological and biological researches. Much of this work I feel will be done on this side of the Atlantic by American students, in magnificent universities, richly equipped with all modern scientific apparatus in the finest laboratories on earth, endowed by the States and independent of the good will of the wealthy.

Before leaving the subject of the pathology and etiology of tumors, I must refer to the Cohnheim hypothesis, which attributes the origin of tumors to a matrix of embryonal cells which lie dormant in the form of small cell-lumps or misplaced islands of cells in the tissues. In order for them to develop, a suspension or diminution of physiological resistance in the tissues (whatever that may mean) must take place in the vicinity of the hypothetical matrix. In the absence of this "matrix of embryonal cells," the possibility of a tumor is absolutely precluded. I suppose the diminution of physiological resistance would be furnished by traumatisms, irritations, inflammations, adverse conditions of life, such as climate, poor environment, hereditary encumbrances, old age, etc.

If the existence of a matrix of misplaced embryonal cells is ever supported by scientific proof, it will be found in the form of "rests," "vestiges," or rudimentary organs. These have great significance when viewed from the developmental standpoint. Here as elsewhere in the social and natural sciences, evolution will be the torchlight that will lead us out of darkness and ignorance into light and knowledge.

Let us now turn to the practical side of our subject and see what we can do in the way of treatment of malignant tumors. In the middle ages, when it was believed that an omniscient and watchful Deity distributed cancers among sinners as in his wisdom he saw fit to do, prayers were the logical remedy to be employed, and we have records of cures by this means. We also have certificates of cures of cancer by the more modern Christian Science, and the pamphlets sent out by the promoters of health resorts and of many natural springs contain plain statements of cases of cancer which were cured by the air and the waters of these springs. Several times in my life, as a surgeon, patients have come to my office bearing scars upon their bodies which they claimed were the scars of former cancers that had been cured by the waters of some springs or by the pastes of some quack. In every single instance a careful investigation proved to me that the scars were those of syphilis or some more benign ulcer that had healed up rather in spite of, than by virtue of, the treatment they had undergone. The ignorant laity, as well as designing fakirs, are only too prone to call any chronic ulcer which does not yield to ordinary treatment a cancer. They do not know that the diagnosis of cancer is often very difficult and sometimes uncertain after all has been done that science offers. I have never seen a spontaneous cure of a cancer, nor have I ever seen one cured by medication of any kind whatsoever. The recently-introduced methods of inoculation with erysipelas and other toxines, as well as the serumtherapy of cancer, have now been amply tried and found wanting. I myself tried these methods on some cases which, in my opinion, were inoperable, but could never record a permanent improvement, and, of course, a cure was out of the question. Where we find reports of favorable results of the inoculation treatment by good surgeons, we will usually find a statement that the pathologist found the tumor to be a spindle cell or a small round cell sarcoma. As above stated, the microscopic examination is not capable of positively recognizing or differentiating these forms of sarcoma from granulomata, or even from benign tumors which have their origin in the lymphatic or connective tissues. The pathologist was in error, and it is probable where a tumor recurs after an operation, in or near the scar, that it is a granuloma, a keloid, or a benign fibrous or myxomatous tumor, or perhaps simply a thickened cicatricial mass, such as we sometimes find in the subcutaneous tissues after operations. These growths sometimes require months and even years for their final absorption.

I do not wish to disparage the serotherapy or the inoculation therapy, and not even the injection of pyoktanin or alcohol, or any other chemical into the substance of inoperable cancers. These methods, or new ones more or less like them, should all be tried before they are condemned. They are deserving of elaborate trial, as we cannot predict their effects a priori. And in inoperable cases the attending surgeon must, at least for psychical effect, if for no other, do something to encourage the unfortunate patient in order to keep him from despondency or suicide.

I must insist, however, that in all operable cases the surgeon has no right to temporize or put off the operation for a period of more than 4 or 6 weeks, and even that short time may injure the chances of a successful operative cure. Many of you will now ask how can we draw the line between operable and inoperable cases. This, I will answer, is a purely relative matter, and what is inoperable for Dr. A. is operable in the opinion of Dr. C., and Dr. C.

will perhaps consent to do the operation at once, and Dr. D. will even urge it and hold out good prospects of a successful operation, and, though a cure can never be promised, he will encourage the patient and inspire hope to such a degree that he will demand to be given the only chance of a cure which we have to offer.

The question as to the curability of cancer is finally and completely settled. Cancer, being a local disease in the beginning and spreading in the well-known way through the tissues by way of the lymphatics and the bloodvessels, must be quite curable when it is possible to remove all the offshoots from the primary nodule. Clinical evidence and the observations of all surgeons who have been in the work for over fifteen years bear out this truth. Formerly when cancer was regarded very generally as a constitutional affection the operations were done with a view to merely prolong the patient's life for a short time, and a cure by operation alone was looked upon as hopeless. Thus the operator did his work in a half-hearted way, and feeling that it could not achieve a complete cure, he did not feel justified in doing the extensive and formidable operations which we do nowadays. In the operation for the removal of the cancerous breast as it has been developed, and as I do it in every instance, the wound is larger in area than the wound made for the exarticulation of the lower extremity at the hip. Some of our most careful and conscientious surgeons require as much as three or four or even five hours for the complete operation of a cancerous breast. In my opinion the surgeon must be prepared to do a complete operation no matter how long it may take to do so, because upon the perfection and detail of the extirpation of all the diseased tissues depends the result. In one case I removed the breast, all the pectoralis major muscle, all of the minor, a good part of the fascia of the external intercostal muscles, all of the contents of the axilla except the nerves and vessels and the clavicle, together with a large bunch of supraclavicular glands and soft parts. The lady has had no return. The operation was done over six and one-half years ago. In cases of cancer of the breast I have seen slightly more than half free from return three years after operation in something more than two hundred and fifty cases. Of the last one hundred cases done over three years I have evidence of only thirty-one recurrences. I may as well say here that a recurrence must be treated in the same manner as the primary tumor, by immediate and radical extirpation, and should there be a return, this must be attacked in the same way until the case is cured or becomes plainly inoperable by the involvement of inaccessible parts. There again I am reminded that what to one surgeon might appear inaccessible to another might be perfectly within reach of the knife and scissors. Only he who has seen good results follow extensive and complete extirpation of whole regions of the body, large parts of the intestinal tract, etc., will have the inclination to do this line of work. Each surgeon soon learns the limitations of his skill, if he is convinced of the usefulness of extensive extirpations of cancerous growths. Every once in a while he will have a facer when a patient dies of shock following one of his efforts to save a life. Deaths of this kind remind us of our limitations in such forcible and persistent manner that we are made to feel the responsibility of greatly endangering human life, even when that life is tainted by a destructive cancer. I remember at this moment, some sleepless nights spent in going over and over in my restless brain the steps of a fatal operation, trying to find in what detail of the work I might have acted differently, perhaps better; how I might have saved the patient a few drops of the impoverished blood, had I acted otherwise; how it might have been better if perhaps no morphine had been injected before the anesthetic was given. Thus a thousand more or less fantastic ideas rack one's brain, keeping away sleep, while the body is tossed from side to side until, the problem still unsolved, sleep finally comes to the relief of the exhausted organism. A few nights of this kind will dampen the ardor of even the most enthusiastic operator, and, unless nature has fitted him for the work by having given him strength and such nerves and organs of sense as will enable him to stand the ordeal of hard work in the daytime and a sleepless night, now and then, he will abandon surgery and devote himself to some other line of work in the profession. There seems to be no danger then that surgeons will often go too far in their operations for the extirpation of cancers by the knife. Until recent years under the influence of antiseptic methods operations were entirely inadequate because they did not go far enough. But now we have arrived at a stage in surgery where we actually operate for the cure and not for the amelioration of cancer cases. Thus it will be seen that in recent years the object of operations for cancer has changed from a mere attempt at amelioration of suffering and prolongation of life to an attempt at a radical cure. Let us now look at the limitations of the operative work. In examining a case of cancer the first question we must answer is, whether or not there is any chance of curing the disease. The examiner will direct his attention to the lymphatics leading into and away from the diseased area with a view to detect any deposits or indurations in the same. He will also direct his attention to the lymphatic tissues some distance away from the cancerous growth, which may have become infected, though there be no visible or tangible change in them. Having determined these points you can see that before beginning the operator knows what he will have to do. It will be his duty not only to remove all the plainly visible infected tissues, but also as much more of the surrounding tissues as in his judgment may be the seat of microscopic nodules or cell nests. If the operator deliberately sets out to cure a case of cancer he will have made up his mind to remove all of the diseased and suspicious tissues, and as you can readily see the operation will be a much more extensive procedure than was ever formerly undertaken. Just for example I will mention two of the most important points. In a case of cancer of the breast, if the tumor has grown outwardly, so as to affect the skin, it will be necessary to remove a large portion of it, because cancer is known to spread most rapidly in the subcutaneous lymphatic spaces. In a case of cancer of the tongue, or again of the breast, if the cancer has invaded one or more of the adjoining muscles it will be necessary to remove all of the affected muscle from its insertion to its origin and even the fascia surrounding the muscles must be entirely removed. The cancer cells are known to travel in the large lymphatics of the muscle and the perimysium with great ease and rapidity ; and if there is to be anything like certainty of cure these structures must be carefully and entirely removed. It is a good rule rather to remove more tissue than less even at the risk of leaving impaired and crippled mobility of parts involved. You will judge from what has been said that an operation performed with a view of curing a patient of a cancer will become a much more serious one than the operation which simply aims at prolonging a patient's life by getting rid of the main trouble.

If a radical operation is judged to be anatomically possible by the operator, the question arises, what is the prognosis, *quoad vitam?* in other words, what per cent. of chance has the patient to survive? The question as to the functional disturbance must also be taken into account; the surgeon must consult with the patient about the matter; both of them must consider the proposition and must answer the question, Is life worth living when certain functions are impaired or lost? In answering this question we must remember that we are dealing with a disease which is usually rapidly fatal, and if it involves certain localities, as, for instance, the throat, or the pylorus, or the bladder, is often the cause of extreme suffering before it produces death. Some of these cases are also not only offensive to their surroundings, but to themselves, by the intolerable odors which they emit.

My object in this paper is to advocate early and radical interference by operative extirpation, and to combat the tendency of some surgeons to operate only on quite simple cases. I cannot admit that the selection of only simple cases for operation is a proper point of view. No doubt the only way to get good statistics is to avoid mortality after one's operations. This desire to avoid mortality always strikes me as a selfish and unprofessional, yes, even a mercenary aim. What are you going to do with the poor fellow who is not operated on because the surgeon fears the possibility of a fatal issue? Has he not a right to demand of you that you give him his only chance? I cannot see that we have a right to refuse an operation if the disease can be removed, unless the outcome would be a hopeless functional result or almost certain death at the operation or soon after it.

Knowing that cancer is curable if totally extirpated, and knowing that it is fatal if not totally removed, I have arrived at the following method of dealing with patients who seek aid from me when they are afflicted by a cancer:

1. When the case is clearly curable and the danger is slight, then I feel it my duty to urge the operation and insist on an immediate performance of the same, and represent the prospects of a cure as highly favorable.

2. When there is considerable involvement of the lymphatics I do not strongly urge the operation, but tell the patient that the chance of a cure, though small, still exists, and in fact feel it a duty to make the attempt at a radical extirpation.

3. When the danger of an operation is very great, but where I still think the removal of all involved tissues can be completed,—I mean by that where the operation is anatomically thinkable,—I present the grave dangers of an immediate death on the operating table, and though holding out but little hope of a cure and only a probable prolongation of life, I still give enough encouragement to inspire the patient with some expectation of being benefited. As a rule, I find that these patients will decide to have the operation done.

4. Finally, in anatomically impossible cases I do not think it is justifiable to refuse to operate in some fashion if after knowing the truth as to the hopelessness of the condition a patient demands that an operation be done. In these cases the psychical effect of even an imperfect partial removal of the offensive cancer sometimes is very beneficial, and puts off for months the inevitable morphine syringe which we are compelled to use during the last weeks.

The time allotted to me will not permit of taking up different forms of cancer in different regions of the body, and I have chosen to present to you the rules that should govern us in only one special region. Let us consider now the management of cancer of the stomach, one of the most frequent and most surely fatal forms of the disease. In the management of gastric cancer two different objects are to be aimed at, and two entirely different operations must be borne in mind in treating a case. If we can reasonably hope for a cure, then we at once contemplate the excision of the diseased tissue, because if there is a chance of a cure we have a right to undertake a dangerous operation.

The operation will be an excision or resection of the pylorus or of a larger part of the stomach, or even of the whole stomach. If a cure is not thinkable, *i. e.*, is anatomically impossible, then we must perform an operation which will ameliorate the patient's condition by overcoming the stenosis and by preventing starvation. The operation we will choose for this purpose is one of the forms of gastro-intestinal anastomosis, gastro-jejunostomy and gastro-duodenostomy, the two operations which come into practice. I do not intend to discuss the various technical modifications of these two operations, but will speak of the diagnosis and indications for surgical interference. I regard it as most desirable that from time to time an accounting be rendered to the general practitioner about the results of the treatment of cancer by surgical operations.

I am of the opinion that it is the duty of every physician at the present time, to call his patient's attention to the possibility of a radical removal of his disease by an operation. Unfortunately the diagnosis of cancer of the stomach is not always easily made in the early stages.

We must not wait until a tumor can be felt. We must recommend an operation if there are symptoms of chronic stomach trouble in persons of above middle age which point to ulcer or stenosis. An essential pointer is given us if we can detect malnutrition and indigestion accompanied by a perverted chemical action. I refer especially to the absence of hydrochloric and the presence of lactic acid in the gastric juice, together with coexisting signs of retention of contents in the stomach, which would indicate pyloric stenosis. We dare not forget that even when the chemical action of the stomach is normal there may be malignant disease.

In a case in which I made the first total excision of the stomach done in America, the diagnosis of cancer had been made by the attending physician, although a tumor could not be plainly made out. The patient had been carefully treated for several months on the supposition that he had a gastric ulcer. He had a number of hemorrhages of almost pure blood. At the operation I found a most extensive tumor. There was no stenosis, and the pylorus was entirely free from disease. In another case recently operated on the diagnosis of cancer was made by competent physicians without the presence of a tumor, and there was no dilatation of the stomach. At the operation I found an enormously large, far-advanced pyloric cancer, with narrow stenosis. The tumor was adherent to the pancreas and extended up towards the hilus of the liver and involved about half of the minor curvature. You see that it will not do to wait until a tumor can be palpated, and I regret that I sometimes did not operate early enough because I could not feel a tumor.

The exploratory incision is free from danger, and by its means we can make an early diagnosis and sometimes render good service even if we should find a non-malignant trouble. The only case in which harm may be done by the exploratory incision is when we make it upon patients too nearly dead from starvation and cachexia.

Persistent, progressive stomach troubles in persons over forty-five years of age are much rarer than is usually accepted by physicians, unless there is a mechanical foundation for them. This foundation will be found in an existing stenosis or ulcer of a cancerous nature in the vast majority of cases. The "sympathetic" stomach troubles due to gallstones, floating kidney, Bright's disease, or some form of constitutional disease having been excluded, nothing remains for us but the diagnosis of gastric cancer.

The trouble which most commonly is confounded with cancer is simple ulcer. But here, as a rule, we have hyperacidity, hyperchlorhydrosis. But we may console ourselves about this matter, because even in benign ulcer operative therapy is often the only correct, the only successful course. In this direction in recent years good work has been done. Leube of Wurzburg, in 1897, at the congress of surgeons, threw much light on the subject by giving his large experience in one thousand cases of gastric ulcer treated by internal medication. Leube found gastric hemorrhages in 46 per cent. of these cases, and still there was only a mortality of 4 per cent. In a series of 556 cases, six died of uncontrollable hemorrhage, six from perforation and peritonitis. In three-fourths of all his cases of benign ulcer Leube achieved a complete and permanent cure by means of a dietetic and medical treatment lasting four or five weeks; one and one-half per cent. remained unimproved and two and four-tenths per cent. died. Leube thinks that the remaining 21 per cent. would have been cured by a repetition or continuation of the treatment.

Leube, who is not a surgeon, recognizes the following indications for surgical treatment :

1. Repeated hemorrhages.

2. Severe pain and frequent regular vomiting, which persists after medical treatment and dieting.

3. Perforation of an ulcer of the stomach.

We are glad to record the fact that a most competent medical authority recognizes that surgery is indicated even in benign ulcers, and I hope you will agree that surgery must not be appealed to as a last resort. The rank and file of the profession must give up the idea that surgery is a last resort, and familiarize themselves with the idea of calling the surgeon early. It is just as easy to prescribe a surgeon for a patient as a dose of morphine.

Billroth said that there must be more surgery in the practice of medicine, and when correctly understood this thought is entitled to great weight. I am perfectly willing to be used as a tool by the physician, and I think all normally-constituted surgeons will recognize that the physician's calling is more arduous than that of the surgeon, because really the diagnosis of internal diseases postulates a much more scientific education and training than is essential to the surgical specialist.

The most important indication for surgical interference is a suspicion that the case is one of cancer. Even where the suspicion of cancer is not pressing the exploratory operation is indicated. The smaller the cancerous tumor the more chances will we have of a radical cure by its removal. I should consider it a most urgent indication to open the abdomen if a competent physician suspected cancer in a patient whom he had carefully examined and treated for a month or two. Should we find a simple ulcer, after opening the abdomen, no harm will have been done, and the case will have been cleared up, and either medical or surgical treatment can be rationally and satisfactorily carried out. When in doubt, operate !

In looking over the statistics of operations in gastric cancer, the important fact is found that we can now furnish the proof that patients from whom we have removed parts of the stomach, yet even the whole stomach, for cancer, are enabled to digest and assimilate food and keep well nourished.

I will quote the statement made by the great surgeon, Kocher, in summing up his entire experience in the surgical treatment of cancer of the stomach by resection. Up to the summer of 1898 he had done 57 excisions of cancers of the stomach. There were then twelve of them living. Four more had lived in a good state of health over three years. One man was drowned while bathing, and in perfect health. One woman died of a cicatricial stenosis, and Prof. Langhans, who made the autopsy, could find no trace of a return. One other man died after a year's good health in an insane asylum. One woman died four years after the operation from an unknown cause, but Prof. Kocher thinks it possible that she had cancerous glands somewhere in her abdomen. Besides these four cases there are five permanently cured patients. These are in the enjoyment of perfect health. One of them was operated on in 1888, one woman in 1893, one man and one woman in 1895, and one man in 1896. These patients are in excellent condition, well nourished and contented.

My own experience, though very large, does not show results as good as that of Kocher. My work was done in St. Louis, Mo., and in private practice to a large extent, and the cases were not in as good condition as those of Kocher, whose work was mostly done in a public hospital, in Bern, on patients who were under good control. Out of twenty-one resections I have only two deaths following immediately upon the operation. All the others survived the operation, and I may say all of them were benefited to some extent, and one lives to-day from whom I removed a pyloric cancer as large as a child's head, seven and one-half years ago. The man is now sixty years of age and works a forty-acre farm. Thirteen died within one year from a return of the cancer, and seven lived from one and a half to four years. (Of these two are still in good health.)

From Kocher's statistics it is clear that the time has come in which we may speak of permanent cures in cases of cancer of the stomach, the most dreadful of all the forms of cancer that we know of, because if left alone, in many cases the patient is condemned to actual and hopeless starvation. A decided improvement in the mortality is also noticeable. Kroenlein lost three out of his first four operations, and only lost two out of the last twenty. The same can be said of almost every surgeon who has kept up the good work of trying to cure cancer of the stomach in the fact of an early appalling mortality. If the general practitioner will send his cases for early operation we may expect a mortality of less than ten per cent. If, however, the extreme skepticism about the success of operations prevails and the unfortunate patient seeks surgical relief after cachexia has begun, we will have a high mortality and bad results.

I have made it a rule in recent years not to attempt a resection on weak patients, and limit my operation to a relief of the stenosis by a gastro-enterostomy. In patients who are comparatively strong, and in whom there is a localized and movable tumor, the radical operation is indicated.

Since Schlatter, in Zurich, successfully performed his total excision of the stomach, a number of these operations have been done by others, but the results prove that suitable cases for this operation are probably not common.

In conclusion let me say to you that if as a result of this address some cancer cases will be driven to seek relief by the only rational means we have to offer, namely, by careful and complete surgical eradication, I shall feel that my effort has been rewarded. Should a few cases be radically cured and twice as many killed by the operation, all will have derived more benefit than if none had been operated on. For those who are killed by the operation are saved from a most horrible slow death. I am perfectly conscious that in thus strongly advocating operation not only in simple, but in advanced cases of cancer, I will meet some adverse criticism. I am consoled by the thought that a valuable victory cannot be gained without first having a fight.—*Medical Sentinel.*

---

THE American Association of Obstetricians and Gynecologists will hold its twelfth annual meeting in the Assembly room of the Denison House, Indianapolis, Ind., Tuesday, Wednesday and Thursday, September 19, 20 and 21, 1899.

TYPHOID FEVER AMONG OUR SOLDIERS IN THE LATE WAR.

Dr. Victor C. Vaughan (*American Journal of the Medical Sciences*, July), who was appointed in August, 1898, by Surgeon-General Sternberg, in conjunction with Major Walter Reed, United States army, and Major E. O. Shakespeare, United States army, on a commission to inquire into this subject, says:

"To summarize concerning the so-called protracted malarias reported by the regimental surgeons, we will say that, in our opinion, practically all of these were typhoid fever, and the following are our reasons for this opinion:

"1. The uneven distribution of the so-called malaria among regiments encamped side by side gives us cause to suspect that these were not malaria.

"2. Some of the surgeons who failed to record their cases as typhoid state in their comments that typhoid fever prevailed in the regiment. One illustration of this may be of interest. From April to November there were in the Sixth Ohio, according to the recorded diagnoses in the monthly reports, only two cases of typhoid fever. Notwithstanding this fact, the acting assistant surgeon in charge at that time writes in the October report the following statement: 'The health of the command is improving. The prevailing disease that has caused the sickness we have had is typhoid fever.' And yet the September report showed only one case of typhoid fever, and the October report showed only one case, and this one case was one and the same patient carried from the August report on to the reports for September and October.

"3. The results of several hundred blood examinations show that malaria was a very rare disease among the troops that remained in the United States.

"4. Malaria as it exists in this country is easily controlled by moderate doses of quinine. All the so-called protracted malarias in our camps were treated with large doses of quinine and were not improved thereby. Consequently, we must conclude that the diagnosis given these cases was erroneous.

"5. The mortality of the so-called protracted malarias corresponds with the mortality of typhoid fever, and furnishes most positive proof that these cases were not malarial."

As regards the question of changing camps, Dr. Vaughan says:

"The influence of change in location of camps upon the continuance of typhoid fever is exceedingly interesting. We have not investigated this subject as thoroughly as we hope to do. At present, however, I think that we can give the following conclusions:

"(*a*) A regiment thoroughly infected with typhoid fever does not lose the disease when it changes its locality, even when it goes to a perfectly unobjectionable site and leaves all its sick behind. Evidence of this is shown in the history of the whole of the Second Division of the First Army Corps when it moved from Chickamauga to Knoxville. At Chickamauga Park this division became thoroughly saturated with typhoid fever. After reaching Knoxville, and notwithstanding the fact that the camp site was probably as fine as any in the world, the water supply unobjectionable, the nature of the soil suitable for the digging of sinks, surface drainage good; notwithstanding all of these advantages, typhoid fever prevailed more largely than it did at Chickamauga. It is altogether probable that the clothing, blankets, and tentage of the men became thoroughly infected with the typhoid bacillus. When a regiment becomes saturated with typhoid fever change in location alone is not sufficient to get rid promptly of the disease. We believe that it will be necessary in such a case thoroughly to disinfect clothing, blankets, tentage, etc.

"(*b*) If a regiment be moved before the infection has become marked typhoid fever may disappear, or at least decrease.

"(*c*) Apparently a sea voyage of some days or weeks might rid a command, not widely infected with typhoid fever, of the disease. We have some illustrations of this in the troops that went to Cuba. For instance, about half of the First Volunteer Cavalry went to Cuba while the other half remained at Tampa. Before the departure for Cuba, and while the regiment was all together, typhoid fever appeared, and its prevalence was confined to those companies that went to Cuba. Apparently this part of the regiment lost its typhoid fever on its way to Cuba, while the part that remained behind became badly infected.

"(*d*) A regiment thoroughly infected with typhoid fever does not lose the disease or lessen the number of cases after a short voyage at sea. This was illustrated by the Nineteenth Infantry, which, after becoming thoroughly infected with typhoid fever, was transported to Porto Rico. The progress of the disease apparently was not in the least retarded by this voyage."—*New York Medical Journal.*

---

### MICHIGAN MEDICAL LAWS.

A law recently enacted in Michigan classes with idiots and insane those suffering from uncured syphilis and gonorrhea as incapable of marriage. They are not reckoned with idiots and insane, however, as regards responsibility, for it provides that "any person who has been afflicted with syphilis or gonorrhea and has not been cured of the same, who shall marry shall be deemed guilty of a felony, and upon conviction thereof in any court of competent jurisdiction, shall be punished by a fine of not less than $500 or more than $1,000, or imprisoned in the State prison at Jackson not more than five years, or by both such fine and imprisonment, in the discretion of the court." The law also provides that the wife can testify against her husband, and *vice versa,* and removes the privilege of medical secrecy in proceedings for this cause. The enforcement of this law will be noted with interest, and it is possible some important medico-legal question may arise: the questions of the curability and the evidence of cure of these disorders may come up in the courts. Considering the frequency of these disorders, especially gonorrhea, it would seem possible that there is danger of a serious diminution in the number of marriages as a result of such law, and also that it might afford possibilities of blackmail and inconvenient family complications. On the other hand, if it will tend to improve morality and diminish the undeserved suffering that these diseases so often entail, one can only wish the new law success. Another Michigan enactment provides that it shall be unlawful for "any person or corporation except a legally incorporated and reputable college of medicine and surgery having and requiring actual attendance at a course of study of not less than three years of eight months each to issue a diploma or certificate setting forth or implying that the holder thereof is qualified to practice medicine or surgery in any of their branches." This, we take it, applies to the diplomas of the notorious St. Luke's Hospital of Niles, and thus helps in wiping out medical diploma mills. A penal clause is attached to this law which will make it imprudent to violate it, and its enforcement ought to be easily practicable. With its new medical practice act and this measure, Michigan ought to be well protected against bogus diplomates and quacks.—*Journal American Medical Association.*

---

### THE PHALLIC PHASE OF "CHRISTIAN SCIENCE."

Under the new medical act "Christian scientist healers," etc., are, for the first time, given a legal status in Illinois. The act contains the following clause explicitly prohibiting interference with them: "This act shall not apply to any person who ministers to or treats the sick or suffering by mental or spiritual means without drugs or material remedy." Under this act the "Christian scientist" has full right to practice without supervision or control by any authorities. Whatever may be said about this backward step in medical legislation, it must be admitted that unrestricted practice of "Christian science" and faith healing is likely to have some queer developments. It is not strange to find the sexual element cropping up in connection with aberrant religious ideas, the relation between sensual practices and religious delusions among the insane having long been known to alienists. This relation explains the frequent sexual aberrations displayed by many fanatic sects. The prophets, John of Leyden, Knipperdoling and the Oneida community—the original "Christian scientists"—exhibited these very strongly in their acts. This tendency has long been recognized as occurring among people of average normal condition by sincerely religious people. Mr. Spurgeon, in a sermon two decades ago, said: "Let us not forget, too, that excess of spirituality is by a strange but certain law placed next door to sensuality." This phallic phase of religion naturally crops up in "Christian science" literature. A recent issue of a Chicago medical journal contains a "Christian science" article on marital hygiene, by one

Ida E. Chaddock, which to the phraseology of the "Christian scientists" joins directions which resemble those given to the priestesses of Aphrodite. It is to be expected that this literature will appear in greater quantities now that "Christian science," so far as Illinois is concerned, is freed from any restriction.—*Journal American Medical Association.*

---

### TREATMENT OF UTERINE FIBROIDS.

F. H. Martin states: 1. The only sure cure for fibroids is the total removal of the uterus; even then, in 5 per cent. of the cases in expert hands death results from the operation. 2. Conservative methods should be pushed to their legitimate possibilities, i. e., the use of medicine, of electricity, and vaginal ligation of the broad ligament. 3. Fibroids would rarely destroy life if left only to conservative treatment. 4. Electricity should be employed as a remedy for fibroids after the fortieth or fiftieth year, when the tumor is interstitial; it should be employed in all inoperable cases. 5. Vaginal ligation of the broad ligament may be employed as a conservative measure in all bleeding fibroids of whatever age, especially in the few years preceding the menopause. It should be employed in all cases of desperate hemorrhage when life is threatened. 6. Removal of appendages should only be resorted to after the abdomen has been opened for hysterectomy and the latter is found inadmissible.—*The Med. Standard.*

---

### HOW TO TREAT SHOCK.

Dr. R. H. M. Dawbarn, of New York, read a paper with this title, in which he discussed more particularly the prevention of shock. He stated that the chief causes of operative shock were: (1) Hemorrhage, (2) duration of the operation, (3) excessive anesthesia, (4) and loss of vital heat. Since 1891 he has made use of systematic intravenous injections of saline solution, and claimed no originality for the method, except as regards the use of these injections at the right time and at the right temperature. By the "right time," he meant their use before major operations to forestall shock; by the "right temperature" was meant that the saline solution should be as hot as could be borne by the hand, *i. e.,* 120° F. —*Med. Review of Reviews.*

